# Structure and magnetism of ultrathin nickel-iron oxides grown on Ru(0001) by high-temperature oxygen-assisted molecular beam epitaxy

**DOI:** 10.1038/s41598-018-36356-6

**Published:** 2018-12-19

**Authors:** Anna Mandziak, Juan de la Figuera, Sandra Ruiz-Gómez, Guiomar D. Soria, Lucas Pérez, Pilar Prieto, Adrian Quesada, Michael Foerster, Lucía Aballe

**Affiliations:** 1 Instituto de Química Física “Rocasolano”, Madrid, E-28006 Spain; 2 Alba Synchrotron Light Facility, CELLS, Barcelona, E-08290 Spain; 3 Dpto. de Física de Materiales, Universidad Complutense de Madrid, Madrid, E-28040 Spain; 4 Unidad Asociada UCM-IQFR(CSIC), Madrid, E-28006 Spain; 5 Instituto Madrileño de Estudios Avanzados - IMDEA Nanociencia, Madrid, E-28099 Spain; 6 Dpto. de Física Aplicada, Universidad Autónoma de Madrid, Madrid, E-28049 Spain; 7 Instituto de Cerámica y Vidrio (CSIC), Madrid, E-28049 Spain

## Abstract

We demonstrate the preparation of ultrathin Fe-rich nickel ferrite (NFO) islands on a metal substrate. Their nucleation and growth are followed *in situ* by low-energy electron microscopy (LEEM). A comprehensive characterization is performed combining LEEM for structural characterization and PEEM (PhotoEmission Electron Microscopy) with synchrotron radiation for chemical and magnetic analysis via X-ray Absorption Spectroscopy and X-ray Magnetic Circular Dichroism (XAS-PEEM and XMCD-PEEM, respectively). The growth by oxygen-assisted molecular beam epitaxy takes place in two stages. First, islands with the rocksalt structure nucleate and grow until they completely cover the substrate surface. Later three-dimensional islands of spinel phase grow on top of the wetting layer. Only the spinel islands show ferromagnetic contrast, with the same domains being observed in the Fe and Ni XMCD images. The estimated magnetic moments of Fe and Ni close to the islands surface indicate a possible role of the bi-phase reconstruction. A significant out-of-plane magnetization component was detected by means of XMCD-PEEM vector maps.

## Introduction

Magnetic oxides with the spinel structure^1^, such as ferrites, are versatile, low-cost materials with a high electromagnetic performance over a wide frequency range^2,3^. When two different metal cations are incorporated into the spinel structure, the possibility of changing the stoichiometry and the cation distribution allows to tune the electronic and magnetic properties over a large range. In particular, nickel ferrite, NiFe_2_O_4_ (NFO) has attracted interest due to a high Curie temperature of 850 K, good insulating behaviour with a band gap of ∼1.5 eV^4^ and a large exchange splitting. Furthermore, changing the conditions of the preparation method^5^ allows to fine tune its properties, even to the point of ordering the Ni cations in octahedral sites^6,7^ (the Ni cations have a strong preference towards the octahedral sites, i.e., NFO is an inverse spinel). Fascinating properties such as the spin caloric effect^8,9^ have already been reported. However, in order to integrate ferrites into electronic or spintronic devices, it is necessary to fabricate thin films of these materials. And often thin films show novel magnetic properties compared to the bulk material. In the case of NFO, there are reports of increased magnetic moments in the ultrathin limit^10,11^. Spinel thin films are clearly affected not only by size and surface effects but also by growth defects. Such is the case for magnetite, where antiphase domain boundaries^12^ have been proposed^13^ to explain high coercive fields^14^, out-of-plane magnetization, superparamagnetism^15,16^ or unexpected easy-axes^17^. Antiphase-boundaries have been directly observed on NFO films^18,19^.

In its bulk structure NFO shows ferrimagnetic order below 850 K. The magnetic structure consists of two antiferromagnetically coupled sublattices, in each of which the coupling is ferromagnetic. One sublattice is populated by Fe^3+^ (3d^5^, nominal spin magnetic moment 5 *μ*_*B*_) ions located at tetragonal A sites of the spinel AB_2_O_4_ structure, while the other sublattice contains Ni^2+^ (3d^8^, nominal spin moment of 2 *μ*_*B*_) and Fe^3+^ (3d^5^, again with 5 *μ*_*B*_) cations occupying octahedral B sites. Experimentally NFO has a saturation magnetization of ∼2 *μ*_*B*_/f.u. (f.u.: formula unit)^1^ in reasonable agreement with the simple argument that the iron cation contribution in each sublattice cancel each other, and that the net magnetic moment arises thus from the Ni^2+^ cations.

All together, it is considered that the development of growth methods that avoid the formation of antiphase boundaries is a prerequisite for attempting to reproduce the known properties of spinel ferrites in structures of reduced dimensionality such as thin films. Several pathways are proposed to achieve this goal, among them the use of special substrates such as MgGa_2_O_4_^20^, or the control of growth conditions to provide micrometric spinel crystals on surfaces arising from a single nucleus. Using the latter approach we have shown that oxygen-assisted MBE growth at high temperature can lead to spinel oxides such as magnetite or cobalt ferrite films with strongly improved properties^21,22^. In this work, we report on the growth and the structural and magnetic properties of ferrimagnetic Ni_*x*_Fe_3−*x*_O_4_ nanometric-thick and micrometer-wide islands on Ru(0001) single crystals using the same approach. We performed a complete spectroscopic analysis employing surface sensitive spectromicroscopy techniques based on x-ray absorption spectroscopy (XAS) and x-ray magnetic circular dichroism (XMCD), which allow for a precise quantification of the composition, element-specific cationic valencies, and magnetic moments.

## Results

**Figure 1:**
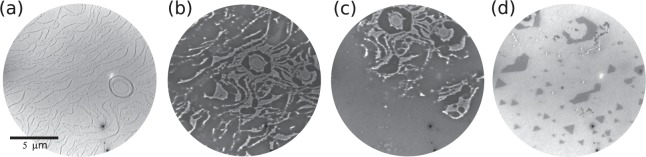
Frames selected from a sequence of images acquired during the growth of NFO at 1150 K at a background pressure of 1 10^−6^ mbar of molecular oxygen. The frames correspond to (**a**) 12 sec, (**b**) 12 min, (**c**) 27 min and (**d**) 49 min after the start of the growth at the rate described in the Methods. The start voltage is 19 V.

NFO thin films have been previously reported by several groups^5,7,9–11,19,20,23–25^. However, films grown by molecular beam epitaxy (MBE) or grown on metal substrates are scarce^26^. As will be shown, growth at a slow rate on a substrate at high substrate temperature can lead to a very low nucleation density and a high crystalline quality of the mixed oxides. Here we deposit nickel and iron on the Ru(0001) substrate while exposing it to molecular oxygen at high temperature. The initial step is to expose the bare Ru substrate to molecular oxygen. Oxygen adsorption is dissociative above room temperature, and forms, at high temperature, a disordered 2-dimensional atomic oxygen gas^27^. A LEEM image of the substrate with oxygen adsorbed on it just after opening the Fe and Ni dosers is shown in Fig. 1a. In addition to the uniform decrease of reflected electron intensity due to the adsorbed gas^28^, many small nuclei of the iron-nickel oxide decorate the substrate steps (curved lines). The Ni and Fe dosing rates were 1:2 with the goal of obtaining stoichiometric nickel ferrite (NiFe_2_O_4_) at rates of the order of 10^−3^ ML/s (were 1 ML is defined as 1.4 10^19^  atoms/m^2^, see Methods). As shown in Fig. 1b–d, islands grow until they coalesce forming a continuous layer covering the substrate, which we call the wetting layer in the following. Continuing the deposition, large islands grow on top of the wetting layer, albeit at a much slower rate, suggesting they grow 3-dimensionally. Thus the NFO growth seems to be similar to the high temperature growth of both iron oxides^29^ and cobalt-iron oxides^22,30^: a flat wetting layer is followed by 3D triangular islands. As we will show next, the similarities extend to the phases we observe in nickel-iron oxides.

**Figure 2:**
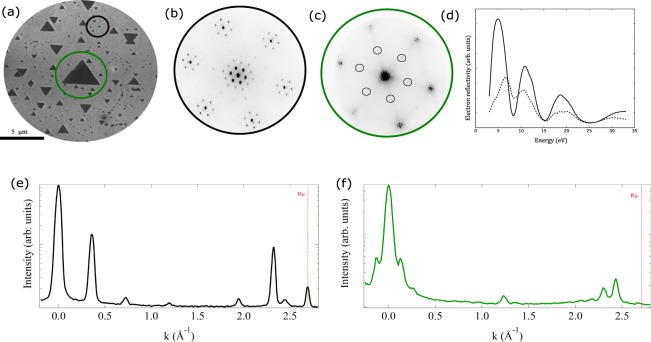
LEEM image (**a**) and corresponding LEED patterns at 35 eV electron energy from different regions of the sample (**b**) wetting layer, (**c**) large triangular island, where some of the 2 × 2 beams are marked with circles. (**d**) LEEM IV spectra (electron reflectivity of the specular beam) from the wetting layer (continuous line) and an island (dashed line). Cross sectional cuts along the moiré pattern at the (**e**) wetting layer (black) and (**f**) island (green), compared to ruthenium (red dashed line).

A microspot low-energy electron diffraction pattern (microLEED) from the wetting layer is shown in Fig. 2b. The pattern corresponds to an hexagonal unit cell with a lattice spacing of 0.31 nm forming a moiré pattern with a periodicity of 2.0 nm (Fig. 2e). The pattern is similar to the one observed for FeO grown in the same conditions^29,31^, with minor differences in the spacings involved. We thus assign it to a similar structure, Fe_*x*_Ni_1−*x*_O. Like in FeO/Ru^31^ the observed pattern is likely to arise from the modulation of the wetting layer due to the formation of a coincidence lattice with the Ru substrate. The lack of additional diffracted spots indicate that there is no further ordering of Fe and Ni, i.e. they form a solid solution. This is unexpected, as the bulk phase diagram indicates a limited solubility of Ni in FeO^32^. From the amount of material deposited at the time needed to close the wetting layer together with the LEED lattice spacing, the wetting layer should correspond to a bilayer of Fe_*x*_Ni_1−*x*_O. This is corroborated by the LEEM IV spectra of the wetting layer, i.e. the electron reflectivity at very low energy for the specular beam^33^, shown in Fig. 2d. It is very similar to the one corresponding to a bilayer of FeO, and very different for a single layer of FeO^31,34^.

The microLEED pattern from the islands shows a 2 × 2 structure (Fig. 2c), marked in the figure with circles, together with very closely spaced satellite spots around each main beam. The spinel spots can in fact also be weakly detected in Fig. 2b due to the presence of small islands in the area where the microLEED was acquired. The distance of the main spots is slighly reduced, corresponding to a spacing of 0.30 nm, while the satellite spots correspond to a 5.6 nm periodicity (Fig. 2f). The 2 × 2 pattern is characteristic of the spinel phase, that along the 〈111〉 direction has a unit cell twice as large as the rocksalt one due to the distribution of iron cations on the (111) planes in the former phase^35^. The 0.30 nm distance in turn corresponds to the oxygen-oxygen distance^29^. The satellite spots are characteristic of the so-called “biphase” surface reconstruction of iron oxides^36^, which we have also observed on magnetite^34^ and cobalt ferrite^22^ on Ru(0001). By knowing the angle between the LEED patterns and the real space images, we find that the edges of the spinel islands are oriented along the [1$\bar{1}$0] direction (which corresponds to the [11$\bar{2}$0] Ru direction). The LEEM IV for an island is also shown for comparison in Fig. 2d.

**Figure 3:**
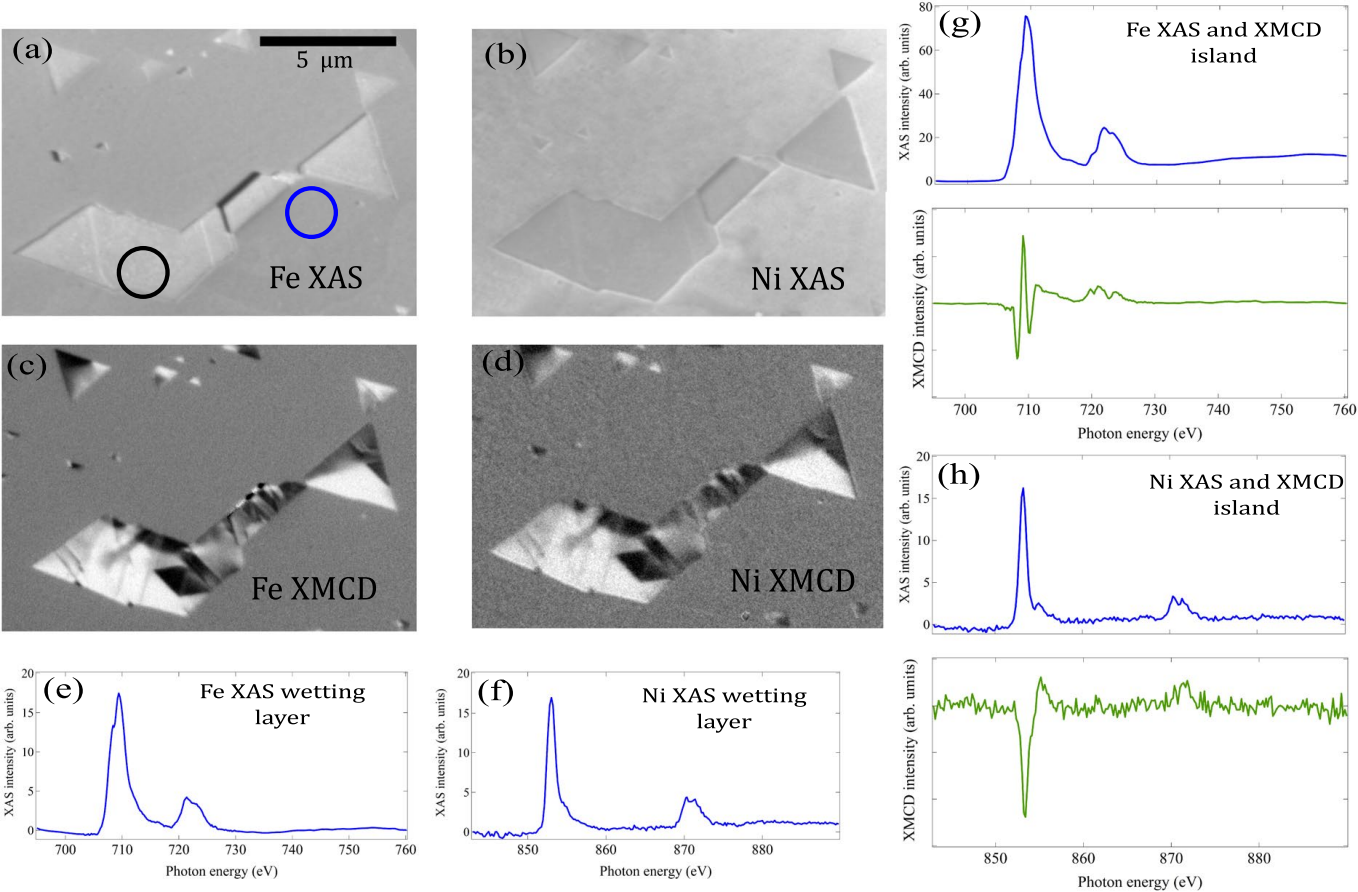
(**a**) Fe and (**b**) Ni XAS-PEEM images acquired at the respective L_3_ edges, divided by the same images acquired at prepeak energies. (**c**) Fe and (**d**) Ni XMCD-PEEM images acquired in the same location. (**e**) Fe and (**f**) Ni XAS spectra from the wetting layer (acquired from the location marked in (**c**) with a blue circle. (**g)** Fe and (**h**) Ni XAS and XMCD spectra acquired from the location of the island marked with a black circle.

The chemical and magnetic properties of our films were investigated by means of XAS and XMCD spectromicroscopy of the Fe and Ni L edges. A XAS-PEEM image at the Fe L_3_ edge is shown in Fig. 3a. Several islands are shown, some of them touching by the edge. The trapezoidal island in the middle shows a shadow on the side opposite to the x-ray beam incidence so its height can be determined: typical heights are tens of nanometers or less (e.g. the island shown in Fig. 3 is 40 nm thick). We note this implies that, unlike the moiré pattern observed on the wetting layer, the bi-phase structure observed on the surface of the islands is a true surface reconstruction and unrelated to the Ru substrate. The Fe islands appear brighter than the wetting layer, suggesting that the islands have more Fe. A similar XAS-PEEM image acquired at the Ni L_3_ edge is shown in Fig. 3b. The same features are seen although much weaker, the contrast is reversed relative to the wetting layer. This suggests that while the islands are clearly richer in Fe than the wetting layer, that is not the case for Ni. To detect the magnetic domains, XMCD-PEEM^37^ images are aquired, substracting XAS images acquired with opposite photon helicites, at an energy close to the maximum of the Fe or Ni L_3_ absorption edge. The islands are shown in Fig. 3c and 3d, for Fe and Ni respectively. Only the islands show black and white regions, corresponding to areas where there is a net component of the magnetization along or opposite the x-ray propagation direction. Thus only the islands show ferrimagnetic domains. The domain structures associated to Fe and Ni match perfectly, although the Ni one is noisier. The black ribbon that corresponds to the shadow in the XAS image presents a reversed magnetic contrast in the XMCD images. This effect is due to x-rays transmitted through the island that hit the wetting layer. But being an XMCD transmission pattern instead of an emission one, the contrast is reversed relative to the latter^38^. The pattern is the same as at the island edge, so the domains extend through the thickness of the island.

From the stack of PEEM images collected at different photon energies, the XAS spectra can be extracted from selected areas of the surface. This is done in Fig. 3e,f for the wetting layer, and 3g,h for the island, respectively. Furthermore, the XAS intensity is proportional to the number of absorbing atoms in the sample. As a consequence, the edge jump, defined as the difference of the intensities above and below an absorption edge, depends linearly on the number of absorbing atoms. Thus the intensity ratio at the minimum between L_3_ and L_2_ edges can be used to provide an estimate of the composition of the area under observation^39^. We find that while the wetting layer has a Fe:Ni ratio of 1:1, on the islands it is closer to 5:1, corroborating that the islands are richer in Fe than the wetting layer. The composition is preserved regardless of the thickness of islands and of the position within the island.

The spectra can also be used to obtain information of the cation oxidation state and site. The spectra of the wetting layer (Fig. 3f) and island (Fig. 3h) are similar to the one of NiO^40^ and of NiFe_2_O_4_ by sputtering^25^, where Ni occupies octahedral sites with an oxidation state of +2 (${{\rm{Ni}}}_{oct}^{2+}$). The L_2_ edge shows a characterictic double peak structure. The Fe spectrum corresponding to the wetting layer is more difficult to interpret, although it is similar to spectra measured in ultrathin FeO^41^ and mixed Co-Fe oxide^42^. The Fe L_3_ edge XAS spectrum from the island is somewhat similar to that of Fe_3_O_4_^43^. However, XMCD spectra often provide a more detailed fingerprint of magnetic oxides^44^. The Fe and Ni XMCD spectra are acquired by substracting pixel-by-pixel the intensity from stack of images acquired with opposite x-ray helicities (Fig. 3g,h), integrated from a single domain area. The Ni XMCD spectrum has a single valley, which corresponds to a single contribution, as expected for ${{\rm{Ni}}}_{oct}^{2+}$^45^. For Fe it presents a three peak structure at the L_3_ edge similar to the one from Fe_3_O_4_:^43,44^ first a valley attributed usually to a contribution of ${{\rm{Fe}}}_{oct}^{2+}$, next a peak which arises mostly from ${{\rm{Fe}}}_{tet}^{3+}$ and finally a valley assigned to ${{\rm{Fe}}}_{oct}^{3+}$. This implies that there is a significant population of Fe^2+^, as expected from the presence of excess Fe relative to the stoichiometric NiFe_2_O_4_ spinel. Thus octahedral positions are occupied by Ni^2+^, Fe^2+^ and Fe^3+^, while the tetrahedral positions are populated only by Fe^3+^. Furthermore, from the Ni XMCD spectra it is clear that the Ni cations have the same magnetization direction as the octahedral Fe^2+^ and Fe^3+^ cations, i.e. they are coupled ferromagnetically. The energies at which the XMCD images shown in Fig. 3c and d were taken correspond to the main peak for the Ni XMCD spectrum and the first minimum of the Fe XMCD spectrum (which corresponds mostly to ${{\rm{Fe}}}_{oct}^{2+}$). Taking together the LEED and the XAS/XMCD information, we suggest that the wetting layer is composed of Fe_0.5_Ni_0.5_O forming a moiré pattern, while the islands have a composition close to Ni_0.5_Fe_2.5_O_4_ and present a bi-phase reconstruction.

**Figure 4:**
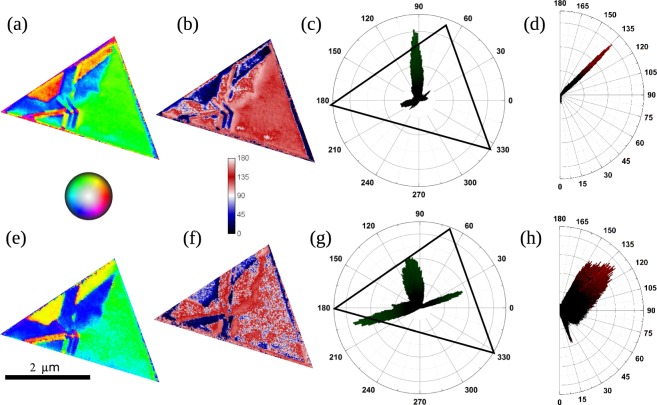
Vector magnetization maps of a Ni-Fe spinel island. (**a**) Fe and (**e**) Ni images of the in-plane orientation, with the colour to angle correspondence shown in the color circle located between them. (**b**) Fe and (**f**) Ni images of the polar magnetization angle from the same island (where 90° indicates in-plane, the color scale is shown between the Fe and Ni images). (**c**,**g**) Distribution of the in-plane magnetization angles, obtained from the data shown in (**a**,**e**) respectively. (**d**,**h**) Distribution of the out-of-plane magnetization angle, obtained from the data shown in (**b**,**f**).

In XMCD the component of the magnetization along the x-ray direction is detected. In order to determine the 3D magnetization vector, at least three different components along non-coplanar directions have to be measured. From images acquired at azimuthal angles 0°, 60° and 120°, and after distortion corrections and transformation from the skew reference frame to an orthogonal one^21^, the vector maps shown in Fig. 4 are obtained for another island of 45 nm thickness. There the in-plane and out-of-plane angle is indicated by the color palette shown between the Fe and Ni images. The magnetic domain size depends on the particular island, but it is often remarkably large, up to several *μ*m^2^. The features observed in the Fe and Ni magnetization maps have a one to one correspondence. The island presents a large domain occupying half of its area, and some intrincate domains on the left portion. A significant out-of-plane angle for the magnetization is detected throughout the island.

Once the magnetization orientation is known and according to the XMCD sum rules^46^ we can estimate the orbital *m*_*orb*_ and spin *m*_*spin*_ magnetic moments from the XAS and XMCD spectra (Fig. 3). If the number of holes per unit cell is known for each cation, the individual values of *m*_*orb*_ and spin *m*_*spin*_ magnetic moments can be obtained as well. In our case the number of holes used for Fe is 4.7 (corresponding to Fe^3+^ ^47^). For Ni^2+^ we have used 2, as for Ni^2+^ in several compounds it is within 10% of that value^48^. The orbital moment is obtained directly while an effective spin moment is estimated, which includes the expectation value of the magnetic dipole operator 〈*T*_*z*_〉. However, the latter is expected to be small for transition metal compounds at room temperature^49^. Self-absorption effects can also be neglected since we are measuring secondary electrons and the probing depth of the order of 1 nm^50^ is well below the x-ray absorption length even for grazing incidence (10 and 20 nm at L_3_ and L_2_ edges, respectively). The moments obtained are *m*_*orb*_ = 0.18 *μ*_*B*_ and *m*_*spin*_ = 0.94 *μ*_*B*_ for Fe and *m*_*orb*_ = 0.13 *μ*_*B*_ and *m*_*spin*_ = 1.13 *μ*_*B*_ for Ni per cation, giving a total moment of 3.4 *μ*_*B*_ per formula unit.

To test the stability of our islands the sample was exposed to air and then reintroduced in the ultrahigh vacuum chamber, softly heated up to 800 K in oxygen atmosphere and re-characterized by means of XAS and XMCD-PEEM. There were no significant changes in the chemical, magnetic or crystallographic structure of the islands.

## Discussion

NFO grown by oxygen-assisted molecular beam epitaxy at elevated temperatures on Ru(0001) results in flat Fe-rich spinel islands surrounded by a mixed NiFe oxide wetting layer with rocksalt structure. The similarity to the growth of both magnetite^34^ and cobalt ferrite^22^ on Ru(0001) should not hide the fact that this is an unexpected result considering the bulk phase diagram of Fe-Ni-O^32,51^: with less than 80% Ni a NiFe alloy should be found in coexistence with FeO with minute amounts of dissolved Ni, while with a larger Ni ratio, the alloy should be in coexistence with Ni-Fe spinels. Our observations highlight the influence of the substrate, as already discussed for Fe oxides^52^. One open question linked to the substrate influence is whether the spinel islands rest directly on the Ru substrate, or whether they lie on the rocksalt oxide wetting layer. We have no proof either way, specially as unlike in ultrathin magnetite^34^, the oxygen lattice in-plane spacing in the wetting layer and the spinel islands differ. Future experiments should be specially designed to address this issue.

The wetting layer does not show any ferromagnetic contrast at room temperature while the islands do. The wetting layer is composed of Fe and Ni forming an “FeO” like structure, i.e. it presents a moiré pattern arising from the coincidence lattice between their rocksalt structure and the Ru substrate. The islands are instead composed of iron-rich nickel ferrite, with the extra iron in the form of Fe^2+^ located at the octahedral sites, while Ni is all in the Ni^2+^ oxidation state at the octahedral sites.

One striking observation is that neither the composition of the wetting layer nor of the islands correspond to the deposited ratio of Fe:Ni of 2:1; from the XAS spectra we estimate an iron/nickel ratio of 5:1 in the islands and close to 1:1 in the wetting layer. Only when we consider the total amount of deposited material (islands plus wetting layer) it is in reasonable agreement with the deposited ratio. This observations of iron-enriched spinel is in line with reports on the oxidation of NiFe alloys, that also produce iron-rich spinel phases^53,54^. We made attempts to increase the Ni ratio in the islands by changing the dosing ratio to Fe:Ni 1:2, but instead of obtaining islands with a higher amount of Ni, the nucleation of 3-dimensional Fe-doped nickel oxide islands was observed (which is in line with the bulk phase diagram^32,51^). Growth at lower temperatures (800–900 K), on the other hand, resulted in segregation into magnetite and metallic Ni. Our results underscore the difficulties to predict the details of the growth of oxides in the ultrathin regime, where the combination of substrate effects, kinetic limitations and thermodynamic factors can give rise to unexpected phases or compositions.

The total magnetic moment estimated from the sum rules is 3.4 *μ*_*B*_ per formula unit. In stoichiometric NFO the net magnetic moment of ~2 *μ*_*B*_ arises from the Ni^2+^ cations as the octahedral and tetrahedral iron cation contributions cancel each other. The larger total magnetization arises in our case from the extra Fe^2+^, which has a moment per atom twice as large as Ni^2+^. In particular, the spin magnetic moments of Fe and Ni were estimated from the sum rules to be 0.94 and 1.13 *μ*_*B*_, respectively. From density functional theory calculations^55^ the Fe and Ni spin magnetic moments are typically reduced when compared with the nominal spin counting values. For example, in Ref.^55^ the spin magnetic moment for Ni^2+^ in NFO is predicted to be 1.6 *μ*_*B*_ instead of 2 *μ*_*B*_, while the spin magnetic moment of Fe^2+^ (in magnetite) is 3.6 *μ*_*B*_. Considering our stoichiometry, we would thus expect 1.4 *μ*_*B*_ per Fe cation and 1.6 per Ni cation. Our values are reduced relative to these predictions. However, we note that the small mean free path for electrons^50^ means the sum rules probe a very shallow surface region. The local magnetization in such region can be affected by the particular surface termination, as has been shown for magnetite^56^. The observation of the bi-phase termination indicates a reconstructed surface. While the exact character of the bi-phase structure is still under discussion^36^, a recent proposal^57^ might imply a reduced magnetization. A definitive conclusion will have to await a detailed atomistic model for the bi-phase reconstruction.

The orientation of the magnetization in the different domains is shown in Fig. 4 as obtained from dichroic images at the L_3_ edges of both Fe and Ni. Both give similar results, the Ni with worse signal to noise ratio. The in-plane orientation of the largest (green) domain is not too far off the side of the island, as also happens with the smaller blue and yellow ones on the opposite side. This suggests that the magnetocrystalline anisotropy is overcome by the shape anisotropy, as expected in a soft magnet like an iron-rich iron-nickel spinel, and recently reported for magnetite islands^21^. However, the magnetization has a well defined out-of-plane component, which is half way from in-plane to out-of-plane (45°). This is quite unexpected, as the shape anisotropy term is expected to drive the magnetization in-plane. In fact, we performed micromagnetic simulations of triangular islands similar in size to the experimental ones using the MuMax3^58^ software and the material parameters for bulk NFO^59^. For such case, the in-plane magnetization should point in-plane along the projection of the magnetocristalline axis, and out of plane with an angle of ±20° with the film plane. As our islands are iron rich with a larger magnetic moment, we expect the shape anisotropy to dominate the magnetization directions, driving the in-plane component to be parallel to the triangles edges -as observed-. But the same effect should also drive the magnetization further in plane, in contrast with the experimental observation. In addition, the domain walls are often remarkably sharp, suggesting a strong influence of contributions such as interface and surface anisotropies or quantum size effects. More work is planned to understand the origin of these magnetization directions and domain patterns. The latter have been observed sometimes in ultrathin magnetite and often in cobalt ferrite islands^22,34^.

It remains to be determined whether the wetting layer mixed FeNi oxide presents antiferromagnetic order and if so, with which Néel temperature. If the wetting layer is magnetically ordered at room temperature, exchange bias effects might play a role in the magnetic properties of the spinel islands, specially if, as discussed before, underlying the spinel layers there is a rocksalt layer.

## Conclusions

Mixed iron-nickel oxides have been grown by oxygen-assisted MBE on Ru(0001) at high temperature. The growth parameters have been optimized by following the growth in real time and real space by means of LEEM in order to obtain large 3-dimensional triangular islands composed of iron-nickel spinels grown on an iron-nickel rocksalt wetting layer of bilayer height. The wetting layer presents a moiré pattern, while the islands have a bi-phase reconstructed surface. Each island is expected to arise from a single nucleus, leading to a low defect density and the observation of large magnetic domains. XAS and XMCD spectromicroscopy reveal that the spinel islands are not stoichiometric NiFe_2_O_4_ but iron rich and that the octahedral positions are occupied by Ni^2+^, Fe^2+^ and Fe^3+^ while the tetrahedral ones are occupied by Fe^3+^.

We estimated the magnetic moment per unit formula, which is larger than for NiFe_2_O_4_ due to the extra iron, but is likely to be affected by the surface reconstruction. Surface magnetization maps have been obtained from XMCD-PEEM images acquired at three different azimuthal angles. An out-of-plane component, unexpected given the bulk magnetocrystalline anisotropy of an iron-rich nickel ferrite, was detected. Together with the observation of sharp domain walls, this points to a sizeable contribution from so far unidentified magnetic interactions.

## Methods

The experiments were performed at the CIRCE end station of the Alba Synchrotron Light Facility^60^. The microscope can work in a pure low-energy electron microscope (LEEM) mode, allowing for microspot low-energy diffraction measurements or dark-field LEEM. By using photons it can be used as an x-ray photoelectron microscope (XPEEM). It can acquire images of the energy-filtered photoelectron distribution in real or reciprocal space, with an energy resolution down to 0.2 eV. The x-ray beam impinges on the sample at an angle of 16° from the surface plane, and the azimuth of the sample can be modified at will. The kinetic energy of the photoelectrons used to form an image can be selected as well. In addition to XAS images, dichroic images can be acquired by measuring the pixel by pixel asymmetry between images with opposite x-ray helicities^37^. Such an XMCD image gives a contrast proportional to the magnetization component along the x-ray direction.

The Ni-Fe oxide thin films are deposited onto a (0001)-oriented Ru single crystal. The Ru substrate is first sputtered with Ar^+^ ions in order to remove previously grown films. Then it is annealed to 1170 K in 10^−6^ mbar molecular oxygen and flashed to 1800 K in vacuum, in order to remove carbon diffusing from the bulk and surface oxide, respectively. Nickel ferrite is synthesized by oxygen-assisted MBE, i.e., co-depositing Ni and Fe onto Ru at elevated temperature (1150 K) in a molecular oxygen atmosphere (1 10^−6^ mbar). Ni and Fe dosers were previously calibrated by measuring the time needed to complete a monolayer (ML) on W(110)^61,62^, 1.4 10^19^ atoms/m^2^. The rates employed for the films presented in this work are for Ni 6 10^−4^ ML/s and for Fe 1.2 10^−3^ ML/s.

Growth is performed during *in situ* observation in LEEM in order to optimize the growth parameters such as substrate temperature, dose rates, or oxygen partial pressure. After the growth process, the sample is cooled down to room temperature in an oxygen atmosphere. The x-ray absorption experiments are typically performed a few days after growth. Before XAS measurement, the sample is briefly flashed to 900 K in 10^−6^ mbar oxygen in order to remove possible adsorbates while avoiding a possible reduction of the film.

## Data Availability

The datasets generated during and/or analyzed during the current study are available from the corresponding author on reasonable request.

## References

[1] Brabers VAM (1995). Progress in spinel ferrite research. In Handbook of magnetic materials.

[2] Harris VG (2012). Modern Microwave Ferrites. IEEE Transactions on Magnetics.

[3] Özgür U, Alivov Y, Morkoç H (2009). Microwave ferrites, part 1: fundamental properties. J Mater Sci: Mater Electron.

[4] Bougiatioti P (2017). Electrical transport and optical band gap of NiFe_2_O_*x*_ thin films. J. Appl. Phys..

[5] Lüders U (2006). NiFe_2_O_4_: A versatile spinel material brings new opportunities for spintronics. Adv. Mater..

[6] Ivanov VG (2010). Short-range B-site ordering in the inverse spinel ferrite NiFe_2_O_4_. Phys. Rev. B.

[7] Iliev MN (2011). Monitoring B-site ordering and strain relaxation in NiFe_2_O_4_epitaxial films by polarized Raman spectroscopy. Phys. Rev. B.

[8] Meier D (2013). Thermally driven spin and charge currents in thin NiFe_2_O_4_/Pt films. Phys. Rev. B.

[9] Shan J (2017). Nonlocal magnon spin transport in NiFe_2_O_4_thin films. Appl. Phys. Lett..

[10] Lüders U (2005). Enhanced magnetic moment and conductive behavior in NiFe_2_O_4_spinel ultrathin films. Phys. Rev. B.

[11] Hoppe M, Doering S, Gorgoi M, Cramm S, Mueller M (2015). Enhanced ferrimagnetism in auxetic NiFe_2_O_4_in the crossover to the ultrathin-film limit. Phys. Rev. B.

[12] Eerenstein, W. *Spin-dependent transport across anti-phase boundaries in magnetite films*. Ph.D. thesis, Rijskuniversisteit, Groningen (2003).

[13] Margulies DT (1997). Origin of the anomalous magnetic behavior in single crystal Fe_3_O_4_films. Phys. Rev. Lett..

[14] Margulies DT (1996). Anomalous moment and anisotropy behavior in Fe_3_O_4_ films. Phys. Rev. B.

[15] Voogt FC (1998). Superparamagnetic behavior of structural domains in epitaxial ultrathin magnetite films. Phys. Rev. B.

[16] Eerenstein W, Kalev L, Niesen L, Palstra TTM, Hibma T (2003). Magneto-resistance and superparamagnetism in magnetite films on MgO and MgAl_2_O_4_. J. Magn. Magn. Mater..

[17] Monti M (2013). Room temperature in-plane(100)magnetic easy axis for Fe_3_O_4_/SrTiO(001):Nb grown by infrared pulsed laser deposition. J. App. Phys..

[18] Datta R (2010). Formation of antiphase domains in NiFe_2_O_4_thin films deposited on different substrates. Appl. Phys. Lett..

[19] Datta R, Loukya B, Li N, Gupta A (2012). Structural features of epitaxial NiFe_2_O_4_thin films grown on different substrates by direct liquid injection chemical vapor deposition. J. Cryst. Growth.

[20] Singh AV (2017). Bulk single crystal-like structural and magnetic characteristics of epitaxial spinel ferrite thin films with elimination of antiphase boundaries. Adv. Mater..

[21] Ruiz-Gómez S (2018). Geometrically defined spin structures in ultrathin Fe_3_O_4_ with bulk like magnetic properties. Nanoscale.

[22] Martn-Garca L (2015). Atomically flat ultrathin cobalt ferrite islands. Adv. Mat..

[23] Johnson MT, G. Kotula P, Carter CB (1999). Growth of nickel ferrite thin films using pulsed-laser deposition. J. Cryst. Growth.

[24] Seifikar S (2014). Structural and magnetic properties of sol-gel derived NiFe_2_O_4_ thin films on silicon substrates. J. Magn. Magn. Mater..

[25] Klewe C (2014). Physical characteristics and cation distribution of NiFe_2_O_4_ thin films with high resistivity prepared by reactive co-sputtering. J. Appl. Phys..

[26] Matzen S (2014). Structure, magnetic ordering, and spin filtering efficiency of NiFe_2_O_4_(111) ultrathin films. Appl. Phys. Lett..

[27] Piercy P, De’Bell K, Pfnür H (1992). Phase diagram and critical behavior of the adsorption system O/Ru(001): Comparison with lattice-gas models. Phys. Rev. B.

[28] de la Figuera J, Bartelt N, McCarty K (2006). Electron reflectivity measurements of Ag adatom concentrations on W(110). Surf. Sci..

[29] Santos B (2009). Structure and magnetism in ultrathin iron oxides characterized by low energy electron microscopy. J. Phys. Cond. Matt..

[30] Martín-García L (2016). Initial stages of the growth of mixed iron-cobalt oxides on Ru(0001). Physics Procedia.

[31] Palacio I, Monti M, Marco JF, McCarty KF, Figuera J (2013). d. l. Initial stages of FeO growth on Ru(0001). J. Phys. Cond. Matt..

[32] Luoma R (1995). A thermodynamic analysis of the system Fe-Ni-O. Calphad.

[33] Flege JI, Krasovskii EE (2014). Intensity-voltage low-energy electron microscopy for functional materials characterization. Phys. Status Solidi RRL.

[34] Monti M (2012). Magnetism in nanometer-thick magnetite. Phys. Rev. B.

[35] Weiss W, Ranke W (2002). Surface chemistry and catalysis on well-defined epitaxial iron-oxide layers. Prog. Surf. Sci..

[36] Parkinson GS (2016). Iron oxide surfaces. Surf. Sci. Rep..

[37] Schneider CM, Schönhense G (2002). Investigating surface magnetism by means of photoexcitation electron emission microscopy. Rep. Prog. Phys..

[38] Kimling J (2011). Photoemission electron microscopy of three-dimensional magnetization configurations in core-shell nanostructures. Phys. Rev. B.

[39] Stöhr, J. & Siegmann, H. C. *Magnetism: From Fundamentals to Nanoscale Dynamics* (Springer, 2006).

[40] Alders D (1998). Temperature and thickness dependence of magnetic moments in NiO epitaxial films. Phys. Rev. B.

[41] Monti, M. *Ultrathin iron oxide films on Ru(0001)*. Ph.D. thesis, Universidad AutÓnoma de Madrid (2014).

[42] Martín-García, L. *Characterization of oxide surfaces and films: real-time growth, interface effects and magnetism*. Ph.D. thesis, Universidad Autónoma de Madrid (2017).

[43] Pellegrin E (1999). Characterization of nanocrystallineγ-FeOwith synchrotron radiation techniques. Phys. Stat. Sol. (b).

[44] Pattrick RAD (2002). Cation site occupancy in spinel ferrites studied by x-ray magnetic circular dichroism developing a method for mineralogists. European Journal of Mineralogy.

[45] Ikeno H (2016). First-principles analysis of x-ray magnetic circular dichroism for transition metal complex oxides. Journal of Applied Physics.

[46] Chen CT (1995). Experimental confirmation of the x-ray magnetic circular dichroism sum rules for iron and cobalt. Phys. Rev. Lett..

[47] Huang DJ (2004). Spin and orbital magnetic moments of Fe_3_O_4_. Phys. Rev. Lett..

[48] Saitoh T, Bocquet AE, Mizokawa T, Fujimori A (1995). Systematic variation of the electronic structure of 3d transition-metal compounds. Phys. Rev. B.

[49] Goering E, Gold S, Lafkioti M, Schütz G (2006). Vanishing Fe 3d orbital moments in single-crystalline magnetite. Europhys. Lett..

[50] Gomes GFM (2014). Magnetic moment of Fe_3_O_4_ films with thicknesses near the unit-cell size. Phys. Rev. B.

[51] Dalvi AD, Smeltzer WW (1970). Thermodynamics of the Iron-Nickel-Oxygen System at 1000C. J. Electrochem. Soc..

[52] Ketteler G, Weiss W, Ranke W, Schlogl R (2001). Bulk and surface phases of iron oxides in an oxygen and water atmosphere at low pressure. Phys. Chem. Chem. Phys..

[53] Wandelt K, Ertl G (1976). Electron spectroscopic studies of the oxidation of Fe/Ni alloys. Surf. Sci..

[54] Greco SE, Roux JP, Blakely JM (1982). Oxidation of the (100) surface of a NiFe alloy. Surf. Sci..

[55] Szotek Z (2006). Electronic structures of normal and inverse spinel ferrites from first principles. Phys. Rev. B.

[56] Martín-García L (2015). Spin and orbital magnetic moment of reconstructed √2 × √2R45° magnetite(001). Phys. Rev. B.

[57] Spiridis, N. Private communication (2018).

[58] Vansteenkiste A (2014). The design and verification of MuMax3. AIP Advances.

[59] Rasic G, Schwartz J (2015). On the origin of coercivity reduction in surface patterned magnetic thin films. Phys. Stat. Sol. (a).

[60] Aballe L, Foerster M, Pellegrin E, Nicolas J, Ferrer S (2015). The ALBA spectroscopic LEEM-PEEM experimental station: layout and performance. J. Synchrotron Rad..

[61] Elmers HJ, Hauschild J, Hoche H, Gradmann U (1994). Submonolayer magnetism of Fe(110) on W(110): Finite width scaling of stripes and percolation between islands. Phys. Rev. Lett..

[62] Kołaczkiewicz J, Bauer E (1984). The adsorption of Ni on W (110) and (211) surfaces. Surf. Sci..

